# Childhood asthma prevalence and risk factors in three Eastern European countries - the Belarus, Ukraine, Poland Asthma Study (BUPAS): an international prevalence study

**DOI:** 10.1186/s12890-016-0172-x

**Published:** 2016-01-14

**Authors:** Grzegorz Brozek, Joshua Lawson, Andrei Shpakou, Olga Fedortsiv, Leonid Hryshchuk, Donna Rennie, Jan Zejda

**Affiliations:** Department of Epidemiology, School of Medicine in Katowice, Medical University of Silesia, Katowice, Poland; Department of Medicine and Canadian Center for Health and Safety in Agriculture, University of Saskatchewan, Saskatoon, Canada; Department of Sport Medicine and Rehabilitation, Yanka Kupala State University of Grodno, Grodno, Belarus; Department of Paediatrics with Children Surgery №1, Horbachevsky State Medical University, Ternopil, Ukraine; Department of Tuberculosis, Horbachevsky State Medical University, Ternopil, Ukraine; College of Nursing and Canadian Center for Health and Safety in Agriculture, University of Saskatchewan, Saskatoon, Canada

## Abstract

**Background:**

The prevalence of asthma and other allergic diseases among children living in Eastern is not well described. Our objective was to estimate and compare the prevalence of asthma, respiratory symptoms and allergic diseases in children in Belarus, Ukraine, and Poland as well as to identify risk factors for these conditions. We also sought to profile and compare children with asthma between locations.

**Methods:**

Data were collected as a part of an international, multicenter, cross-sectional study of childhood asthma: The Belarus Ukraine Poland Asthma Study (BUPAS). Subjects were children aged 7–13 years attending primary and secondary schools in the urban and surrounding rural area of Grodno (Belarus), Ternopil (Ukraine) and Silesia Region (Poland). Physician-diagnosed respiratory diseases and symptoms as well as allergic diseases were ascertained using the ISAAC questionnaire completed by the parents.

**Results:**

In total there were 4019 children from Belarus (rural: 2018, urban: 2001), 4493 from Ukraine (1972; 2521), and 4036 from Poland (2002, 2034). The overall response rate was 76.7 %. Groups were similar in case of gender and age (p > 0.05). Almost all analyzed respiratory and allergic conditions differed significantly between countries including asthma [Poland (rural, urban): 3.5 %, 4.1 %; Ukraine: 1.4 %, 2.1 %; Belarus: 1.4 %, 1.5 %], spastic bronchitis (Poland: 2.7 %, 3.2 %; Ukraine: 7.5 %, 6.5 %; Belarus: 6.4 %, 7.9 %), and chest wheeze in the last year (Poland: 4.8 %, 5.2 %; Ukraine: 11.5 %, 13.0 %; Belarus: 10.7 %, 10.0 %). These differences remained after adjustment for potential confounders. Risk factor associations were generally similar between outcomes. Symptom characteristics of children with asthma between countries were not consistent. The ratio of current wheeze:diagnosis of asthma differed by country: (Rural areas: Belarus: 10.9:1, Ukraine: 17.3:1, Poland: 2.4:1; Urban areas: Belarus: 8.1:1, Ukraine: 7.3:1 Poland: 1.9:1).

**Conclusions:**

The findings show large between-country differences and relatively low prevalence of asthma and allergic diseases in children of Western Belarus and Ukraine. There is evidence for underdiagnosis of asthma in these regions.

## Background

Geographic variation in childhood asthma prevalence has been reported [1, 2] with the global prevalence of asthma and related symptoms has ranged between approximately 2 and 37 % [1]. One difficulty in assessing asthma prevalence is potential differential use of labels for asthma. As such, it is important to symptoms, which do not require a diagnostic label and alternate labels for asthma and the potential of differences in asthma prevalence due to labeling differences.

While some investigation of asthma prevalence has been completed in Eastern Europe, including a regional investigation of countries from Eastern and Central Europe [3] which showed between-country variation in prevalence was more dominant than within-country variation along with some indication of diagnostic labeling explaining differences by region, there has been very little work completed in countries outside the EU including Ukraine and Belarus. Previously, we have described investigations of asthma prevalence separately in Ukraine [4] and Belarus [5] as part of the Belarus, Ukraine, and Poland Asthma Study (BUPAS). In each of these locations, there was some evidence of asthma underdiagnosis [4, 5] Qualitatively, the prevalence of asthma was lower in these countries compared to Western European nations, with possible underdiagnosis occurring. These observations, along with societal changes towards a Westernized lifestyle, which has been associated with higher asthma prevalence, make it likely that the prevalence of asthma will increase in the future. Indeed, we have some evidence suggesting increases of asthma prevalence in Eastern Europe [6]. Thus, asthma may be poised to increase as a public health problem in this region. Identification of reasons for geographic differences and risk factors for asthma, including consistency between regions, in these locations will help develop our etiologic understanding of asthma and may help our future management strategies for children with lung disease.

We further our previous work of BUPAS countries by combining the data from each country, which were based on data collection using the same questionnaires and study design. Specifically, we sought to: 1) compare prevalence of diagnosed asthma, diagnosed spastic bronchitis, respiratory symptoms, and allergic disorders between the three BUPAS countries; 2) To compare the prevalence and strength of association of risk factors for diagnosed asthma, wheeze, and spastic bronchitis between the three BUPAS countries; 3) To analyze the magnitude of urban–rural differences in the occurrence of diagnosed asthma in the three BUPAS countries; and 4) To compare symptoms profiles of children with diagnosed asthma between the three BUPAS countries. While we have previously described asthma prevalence and its determinants in Belarus and Ukraine as part of the BUPAS program, [4, 5] we have expanded on our previous investigations. First, the focus of the current paper is on between country differences, which allow us to make quantitative comparisons between regions. Second, this will be the first time where results from the Polish data are presented. Third, while minor, we focus on a slightly narrower age range than the previous studies in order to be able to include comparative data from all countries.

## Methods

### Study design and study population

Data were collected as a part of an international, multicenter, cross-sectional study of childhood asthma: The Belarus Ukraine Poland Asthma Study (BUPAS). Subjects were children aged 7–13 years attending primary and secondary schools in the urban and surrounding rural area of Ternopil (Ukraine), Grodno (Belarus) and Katowice (Poland). This age group was chosen given that this is generally when asthma prevalence is highest, it allows reasonable responses based on proxy report (i.e., parents), and for comparison, is comparable to ages consistently considered in other studies evaluating asthma prevalence in childhood. Schools were randomly selected from the urban center and the rural region, which included villages where agricultural activity was the primary industry, surrounding each urban area from which 3000 urban children and 3000 rural children were approached. This included 36 schools from urban areas in Belarus (10), Ukraine (11), and Poland (15) and 79 schools from rural areas in Belarus (20), Ukraine (23), and Poland (36). The number of schools included in each region differ due to differences in the number of students attending each school as participation rates were similar. Children from selected schools and their parents or legal guardians were invited to participate in the project. Questionnaires, accompanied by a letter explaining the study objectives and an informed written consent, were distributed to parents/guardians through the schools. Upon completion of the questionnaire, they were returned to the school where they were picked up by the research team. The study protocol was approved by the adequate local ethics committees at the State Medical University of Ternopil (Ukraine), Yanka Kupala University in Grodno (Belarus) and Medical University of Silesia in Katowice (Poland). None of the authors have any competing interests related to this manuscript.

### Study instrument and operational definitions

The questionnaire included standard questions from the International Study of Asthma and Allergy in Children (ISAAC) respiratory questionnaire [2, 7] All questions were translated from English to Ukrainian, Russian and Polish respectively and their syntax was verified by back-translation. The ISAAC questionnaire has been used internationally and validated in many languages [8]. ISAAC questions have been used in Polish and Russian speaking populations previously [8]. The questionnaire included questions about established respiratory and allergic diagnoses, respiratory and allergic symptoms, family history of respiratory and allergic disorders, and socio-economic status. Location of dwelling was classified as urban or rural based on whether the child lived in the city (Grodno, Ternopil, Katowice) or the surrounding agricultural area. This was accounted for by targeting schools in these regions.

The list of analyzed allergic and respiratory diseases included diagnoses established during the child’s lifetime by a physician and reported by subjects’ parents for asthma and spastic bronchitis. Spastic bronchitis is a traditional diagnosis of the region and is used to describe chronic or recurrent asthmatic symptoms such as “wheezy bronchitis”. Respiratory symptoms in the past 12 months were also assessed by report on the questionnaire.

Several variables were considered as potential correlates of respiratory outcomes. Parental asthma, allergic rhinitis, eczema were separately defined by a report of a positive history of physician diagnosed disease by either the mother or father. Current smoking and highest level of education were obtained for each of the mother and the father. Exposure to tobacco smoke was present when at least one family member smoked at home. Additional exposures were assessed by responses on the questionnaire based on the presence or absence of these exposures and included using coal or wood for cooking or heating, smoking during pregnancy, passive smoking during pregnancy, the presence of dog and cat in the home, and dampness at home. Evaluation of health care utilization and morbidity was based on answers to questions regarding medication use for breathing, specialist or general practitioner visits for breathing conditions, completion of spirometry testing, and completion of skin prick testing, all in the past 12 months.

Height (cm) and weight (kg) were reported on the questionnaire. From these measures, body mass index (BMI) was calculated based on the equation: [BMI = weight (kg)/height (m)^2^] [9]. Weight status was classified based on categorization of body mass index (BMI) using values described by Cole et al. [10] which base the cutoff value as the predicted adult (at 18 years of age) equivalent of a BMI of 25 for overweight and of 30 for obese.

### Statistical analysis

All analyses were completed using SPSS 20. Statistical significance was defined by an alpha level of 0.05. Continuous variables were described by arithmetic mean values and standard deviations. Categorical variables were described by frequencies and proportions. Descriptive and comparative analyses were completed after stratification by urban/rural status because of the method of sampling. Statistical significance of between-group differences in mean values was assessed by *t*-test and ANOVA, or nonparametric tests when distribution was not normal. When using categorical variables statistical significance was assessed by means of the independent samples chi-squared test. Initially, comparisons of personal and environmental factors were made between locations and between urban and rural status for each location. Following this analysis, comparisons of condition and symptom prevalence were made between locations and between urban and rural status for each location.

Multiple logistic regression was then completed to investigate the association between report of diagnosed asthma, diagnosed spastic bronchitis, and wheeze in the past 12 months with location and urban/rural status after adjusting for potential confounders. A multiple logistic model was fitted for each health outcome independently. Potential confounders were included based on their previously reported associations with asthma and availability on the questionnaire. For each model, the same fixed set of independent variables was tested to examine their association with the dependent variable and to control for potential confounding: age, sex, parental education, parental history of allergic disease, exposure to tobacco smoke at home, mother smoking during pregnancy, location of dwelling (urban or rural), use of coal or wood for cooking or heating, dampness, and dog or cat at home. The strength of the associations between the independent and dependent variables were quantified by odds ratios and their 95 % confidence intervals (CI). Statistical inference was based on the criterion *p* < 0.05. Interaction between location and each of the personal and environmental factors was tested using the Likelihood ratio test to assess if associations were consistent between locations. These results were also presented by stratified analyses.

Finally, among children with a previous diagnosis of asthma, we compared indicators of asthma severity and morbidity including hospitalization for asthma, visiting a physician for asthma, assessment by spirometry, assessment by skin prick testing and the presence of asthma-related symptoms between locations. This was completed using independent samples chi-squared tests.

## Results

In total there were 4019 children from Belarus (rural: 2018, urban: 2001), 4493 children from Ukraine (rural: 1972, urban: 2521), and 4036 children from Poland (rural: 2002, urban: 2034). The response rate was 79.2 % in Ukraine, 77.8 % in Belarus and 75.4 % in Poland and was similar (>0.05) between urban and rural areas from each region. When comparing differences in the frequency of personal and environmental characteristics between countries (Table 1), there were many statistically significant differences. Among urban and rural dwellers, there were differences between countries for mother’s and father’s education levels, parental history of allergic disease, parental smoking, maternal smoking during pregnancy, use of coal or wood for cooking or heating, presence of mold or dampness, and presence of a cat or dog. However, the differences were not always consistent in direction or magnitude. In addition to this, a lower proportion of urban dwelling children from Belarus were female compared to urban children from Ukraine or Poland and urban dwelling children from Ukraine were older than urban children from Belarus or Poland (Table 1).

**Table 1:** Personal characteristics of the study population by country and location of residence

	Urban			Rural		
	Belarus (*n* = 2001)	Ukraine (*n* = 2521)	Poland (*n* = 2034)	Belarus (*n* = 2018)	Ukraine (*n* = 1972)	Poland (*n* = 2002)
% Female	47.4	50.9	50.9^**^	49.8	51.5	51.8
% Mother with > high school	46.9^*^	54.1^*^	19.2^*, **^	19.7	41.9	13.0^***^
% Father with > high school	38.8^*^	45.7^*^	13.4_*, **_	16.3	32.0	6.8^***^
Obesity status						
% Not overweight or obese	87.1	87.8	86.8	88.3	85.9	88.2
% Overweight	11.5	10.7	11.8	10.0	11.2	10.4
% Obese	1.4	1.5^*^	1.5	1.7	3.0	1.5^***^
% Any parental history of allergic disease	14.7^*^	11.7^*^	30.7^*, **^	8.8	8.0	20.0^***^
% Either parent currently smoking	50.1^*^	50.9	46.5^*, **^	57.8	53.4	43.0^***^
% Maternal smoking during pregnancy	2.5	0.9	7.0^*, **^	2.4	1.1	4.4^***^
% Using coal or wood for cooking or heating	4.8^*^	1.4^*^	10.6^*, **^	28.3	6.8	28.2^***^
% Any mold or dampness in the home	30.5	47.9^*^	34.2^*, **^	29.9	55.8	27.5^***^
% With a cat	34.6^*^	27.9^*^	34.4^*, **^	61.4	64.2	43.9^***^
% With a dog	23.7^*^	17.2^*^	50.2^*, **^	45.6	54.2	55.1^***^
Mean age, years (SD)	10.2 (1.9)	10.4 (1.9)^*^	10.2 (1.9)^**^	10.2 (1.9)	10.2 (1.9)	10.3 (1.9)

^*^ *p* < 0.05 comparing urban to rural within country

^**^ *p* < 0.05 comparing across countries among urban children

^***^ *p* < 0.05 comparing across countries among rural children

The prevalence of asthma differed significantly by country for both urban and rural dwellers with the highest prevalence consistently in Poland (Rural: 3.5 %, Urban: 4.1 %) followed by Ukraine (Rural: 1.4 %, Urban: 2.1 %) and Belarus (Rural: 1.4 %, Urban: 1.5 %). The difference was not statistically significant between urban and rural dwellers in any country. Almost all other respiratory and allergic conditions considered differed significantly between countries for both urban and rural dwellers (Table 2). Similar to asthma, a higher prevalence of allergic conditions and symptoms, with the exception of eczema or skin allergy, was found in Poland compared to Ukraine and Belarus. While differences between countries were statistically significant, the direction of the differences was not entirely consistent when comparing respiratory symptoms. However, diagnosis of spastic bronchitis was consistently lower in Poland compared to Belarus and Ukraine.

**Table 2:** Distribution of asthma, asthma-like conditions, and allergic conditions by country and location of residence

	Urban			Rural		
	Belarus (*n* = 2001)	Ukraine (*n* = 2521)	Poland (*n* = 2034)	Belarus (*n* = 2018)	Ukraine (*n* = 1972)	Poland (*n* = 2002)
Ever diagnosed with asthma	1.5	2.1	4.1†	1.4	1.4	3.5^***^
Asthma attacks in the past 12 months	0.7	0.9	1.8^**^	0.7	0.7	1.2
Woken by asthma attacks in the past 3 months	0.5	0.4	1.4^*, **^	0.3	0.7	0.7
Spastic bronchitis	7.9	6.5	3.2^**^	6.4	7.5	2.7^***^
Ever had chest wheeze	10.2	32.9*	14.7^*, **^	10.7	30.0	11.9^***^
Chest wheeze in the past 12 months	10.0	13.0	5.2^**^	10.7	11.5	4.8^***^
SOB with exercise in the past 12 months	4.0	6.6	4.7^*, **^	3.6	5.8	3.1^***^
Nocturnal SOB in the past 12 months	1.7^*^	1.8	3.5^*, **^	1.0	1.7	2.2^***^
Cough in the past 12 months	58.4^*^	73.4*	68.8^**^	65.1	70.5	67.8^***^
Productive cough in the past 12 months	34.0^*^	46.0	52.5^*, **^	39.4	44.3	42.8^***^
Ever diagnosed with rhinitis	5.3^*^	3.6	22.8^*, **^	2.1	4.4	14.7^***^
Ever diagnosed with eczema	12.6^*^	7.0	12.7^*, **^	8.6	6.8	7.4
Ever diagnosed with any allergy	17.7^*^	12.0	24.3^*, **^	10.7	13.1	16.1^***^
Ever had symptoms of allergic rhinitis or hayfever	3.7^*^	7.9*	20.6^*, **^	1.1	4.7	14.2^***^
Ever had sneezing with congestion or intense runny nose apart from a cold	10.0^*^	14.3*	21.1^*, **^	7.0	9.7	13.6^***^
Ever had skin allergy or itching	15.3^*^	14.1^*^	11.2^*, **^	12.7	11.7^*^	7.1^***^

^*^ *p* < 0.05 comparing urban to rural within country

^**^ *p* < 0.05 comparing across countries among urban children

^***^ *p* < 0.05 comparing across countries among rural children

Table 3 presents the findings from adjusted analyses for report of diagnosed asthma (primary interest), wheeze in the past 12 months (symptom reflective of asthma), and diagnosed spastic bronchitis (a diagnostic label thought to be used in place of asthma in the region). Compared to Belarus, children in Poland were at a higher risk of diagnosed asthma but a lower risk of wheeze in the past 12 months and spastic bronchitis while children from Ukraine were at increased risk of wheeze in the past 12 months. Females were at lower risk of any of the three outcomes with similar strengths of associations. There was a consistent increased risk of any of the three outcomes with the presence of parental history of allergic disease and mold or dampness in the home. Maternal smoking during pregnancy increased the risk of asthma and spastic bronchitis but this was only statistically significant for asthma. While having a dog was inversely associated with all three outcomes, it was only statistically significant for spastic bronchitis. Finally, increasing age was associated with an increased risk of diagnosed asthma but a reduced risk of wheeze in the past 12 months and spastic bronchitis.

**Table 3:** Adjusted ^a^ associations between respiratory health risk factors and outcomes of report of diagnosed asthma, wheeze in the past 12 months, and diagnosed spastic bronchitis

	Diagnosed asthma OR (95 % CI)	Wheeze in the past 12 months OR (95 % CI)	Diagnosed spastic bronchitis OR (95 % CI)
Country			
Belarus	1.00	1.00	1.00
Ukraine	1.32 (0.86–2.01)	1.20 (1.02–1.43)	0.99 (0.81–1.24)
Poland	2.40 (1.67–3.44)	0.42 (0.34–0.51)	0.36 (0.28–0.47)
Sex			
Male	1.00	1.00	1.00
Female	0.70 (0.53–0.92)	0.81 (0.70–0.94)	0.76 (0.64–0.91)
Mother with > high school			
No	1.00	1.00	1.00
Yes	1.15 (0.81–1.65)	0.91 (0.77–1.09)	1.09 (0.88–1.36)
Father with > high school			
No	1.00	1.00	1.00
Yes	0.93 (0.62–1.38)	1.06 (0.88–1.27)	1.11 (0.88–1.39)
Any parental history of allergic disease			
No	1.00	1.00	1.00
Yes	3.01 (2.25–4.04)	1.62 (1.34–1.96)	2.72 (2.20–3.36)
Either parent currently smoking			
No	1.00	1.00	1.00
Yes	1.18 (0.89–1.57)	1.03 (0.89–1.19)	0.95 (0.79–1.15)
Maternal smoking during pregnancy			
No	1.00	1.00	1.00
Yes	2.42 (1.42–4.11)	0.98 (0.61–1.56)	1.50 (0.90–2.53)
Using coal or wood for cooking or heating			
No	1.00	1.00	1.00
Yes	1.01 (0.67–1.51)	1.02 (0.80–1.29)	1.01 (0.75–1.31)
Any mold or dampness in the home			
No	1.00	1.00	1.00
Yes	1.33 (1.01–1.77)	1.35 (1.16–1.56)	1.28 (1.07–1.55)
With a cat			
No	1.00	1.00	1.00
Yes	0.84 (0.62–1.15)	0.93 (0.79–1.09)	1.04 (0.85–1.27)
With a dog			
No	1.00	1.00	1.00
Yes	0.78 (0.57–1.06)	0.94 (0.80–1.11)	0.77 (0.62–0.96)
Residence			
Rural	1.00	1.00	1.00
Urban	0.95 (0.71–1.27)	0.97 (0.83–1.13)	0.98 (0.80–1.19)
Age	1.11 (1.03–1.20)	0.93 (0.90–0.97)	0.95 (0.90–0.99)

^a^Adjusted for each variable in the table

**Fig. 1:**
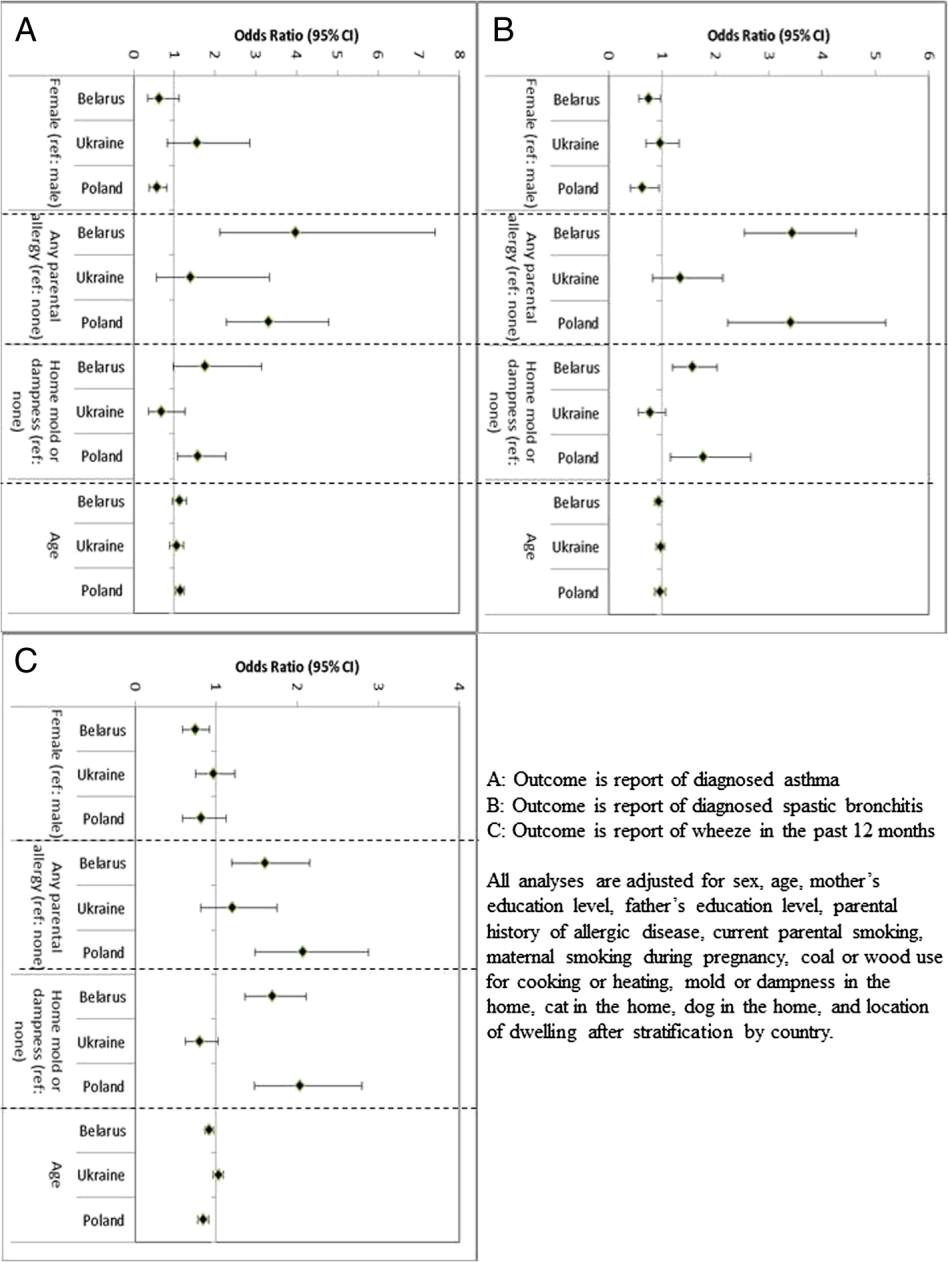
Adjusted associations between personal and environmental characteristics with report of diagnosed asthma, diagnosed spastic bronchitis, and wheeze in the past 12 months. **a** Outcome is report of diagnosed asthma. **b** Outcome is report of diagnosed spastic bronchitis. **c** Outcome is report of wheeze in the past 12 months. All analyses are adjusted for sex, age, mother’s education level, father’s education level, parental history of allergic disease, current parental smoking, maternal smoking during pregnancy, coal or wood use for cooking or heating, mold or dampness in the home, cat in the home, dog in the home, and location of dwelling after stratification by country

We assessed the presence of interaction between country and the personal and environmental variables. When considering asthma as the outcome, there was statistically significant interaction between country and sex (*p* = 0.03) as well as home mold or dampness (*p* = 0.04). When considering wheeze in the past 12 months as the outcome, there was statistically significant interaction between country and home mold or dampness as well as age (*p* < 0.001). When considering spastic bronchitis there was statistically significant interaction between parental history of allergic disease (*p* = 0.004) as well as home mold or dampness (*p* = 0.002). Because of these results, we reran the adjusted analysis after stratification by country and presented the results for the interaction variables in Fig. 1. In general, we found similar associations in Belarus and Poland and weaker, non-statistically significant associations within Ukraine.

Finally, we profiled the clinical characteristics of children with asthma and compared these by rural–urban status and by country (Table 4). When comparing characteristics of children with asthma by country, among rural dwellers, there were differences by country for diagnosis of rhinitis, rhinitis symptoms, taking breathing medication in the past 12 months, using asthma medication in the past 12 months, and being hospitalized for breathing troubles in the past 12 months (Table 4). Among urban children with asthma, differences by country existed for diagnosis of spastic bronchitis, diagnosis of rhinitis, diagnosis of any allergy, ever had a skin allergy or itching, using asthma medication in the past 12 months, being hospitalized for breathing troubles in the past 12 months, and having spirometry testing in the past 12 months (Table 4). Finally, to further consider some issues that may be related to diagnosis, we calculated the proportion of children with wheeze in the past 12 months who had a diagnosis of asthma. Belarus and Ukraine had lower proportions of those with wheeze in the past 12 months having an asthma diagnosis than in Poland for both rural (Belarus: 8.4 %, Ukraine: 5.5 %, Poland: 29.0 %) and urban regions (Belarus: 10.9 %, Ukraine: 12.1 %, Poland: 34.7 %). Related to these results, we considered the ratio of (current wheeze):(previous diagnosis of asthma). In rural areas this ratio was 10.9:1 in Belarus, 17.3:1 in Ukraine, and 2.4:1 in Poland. Similar patterns were observed in the urban areas (Belarus: 8.1:1, Ukraine: 7.3:1 Poland: 1.9:1).

**Table 4:** Distribution of symptoms, allergic conditions, health care utilization, and morbidity by country and location of residence among those with a previous diagnosis of asthma

	Urban			Rural		
	Belarus (*n* = 30)	Ukraine (*n* = 54)	Poland (*n* = 84)	Belarus (*n* = 29)	Ukraine (*n* = 27)	Poland (*n* = 70)
Asthma attacks in the past 12 months	50.0	42.3	42.7^*^	51.7	37.0	26.9
Woken by asthma attacks in the past 3 months	33.3	16.3	35.5^*^	24.1	17.4	16.9
Ever diagnosed with spastic bronchitis	76.7^*^	58.3	38.1^**^	48.3	53.8	37.9
Chest wheeze in the past 12 months	73.3	70.4^*^	45.5	62.1	44.4	40.9
SOB with exercise in the past 12 months	46.7	53.7	36.6	37.9	44.4	24.6
Nocturnal SOB in the past 12 months	30.0	23.1	37.3^*^	24.1	29.6	22.4
Cough in the past 12 months	83.3	90.7^*^	92.7	86.2	74.1	88.1
Productive cough in the past 12 months	80.0	70.0	67.1	58.6	63.0	62.5
Ever diagnosed with rhinitis	43.3^*^	31.9	76.0^*, **^	13.8	25.0	55.9^***^
Ever diagnosed with eczema	56.7^*^	30.6	37.1	10.3	37.5	24.6
Ever diagnosed with any allergy	80.0^*^	46.0	66.2^**^	41.4	68.0	49.3
Ever had symptoms of allergic rhinitis or hayfever	50.0^*^	49.1	61.4	13.8	60.0	48.5‡
Ever had sneezing with congestion or intense runny nose apart from a cold	76.7	66.7	65.1	62.1	77.8	55.2
Ever had skin allergy or itching	50.0	23.1	28.6^**^	37.9	44.4	21.7
Ever used medication for asthma	86.7	42.0	65.8^**^	93.1	55.6	60.3^***^
Hospitalization due to breathing troubles in the past 12 months	20.0	28.8	6.0^**^	20.7	20.0	4.8^***^
Visited a doctor for breathing troubles in the past 12 months	60.0	45.3	50.6	51.7	55.6	41.2
Spirometry testing in the past 12 months	43.3	16.7	26.2^**^	24.1	18.5	37.1
Skin prick testing in the past 12 months	30.0	20.4	22.6	20.7	18.5	31.4

^*^ *p* < 0.05 comparing urban to rural within country

^**^ *p* < 0.05 comparing across countries among urban children

^***^ *p* < 0.05 comparing across countries among rural children

In general, urban children with a report of doctor diagnosed asthma had a higher prevalence of conditions present than rural children. In Belarus we found urban children with a report of doctor diagnosed asthma to have a higher proportion of spastic bronchitis and allergic conditions or symptoms. In Ukraine, urban children with a report of doctor diagnosed asthma had a higher prevalence of wheeze in the past 12 months and cough in the past 12 months. In Poland, urban children with a report of doctor diagnosed asthma had a higher prevalence of asthma attacks in the past 12 months, had been woken by asthma attacks in the past 3 months, and had nocturnal shortness of breath in the past 12 months.

## Discussion

While asthma prevalence was higher in Poland compared to Belarus and Ukraine, spastic bronchitis diagnosis, an alternative to asthma diagnosis in some countries, was higher in Belarus and Ukraine compared to Poland. While chest wheeze in the past 12 months was lower in Poland than in the other two countries and report of diagnosed rhinitis and rhinitis symptoms were higher in Poland, there were inconsistent patterns in symptom prevalence. With the exception of the country of residence and its association with the outcomes considered, the associations between suspected asthma risk factors and asthma were generally consistent between locations where there was an increased risk of asthma associated with parental history of allergy and presence of home mold or dampness along with an inverse association with sex. Our results suggest that differences in diagnostic labeling may be occurring. Symptom characteristics of children who had a report of a diagnosis of asthma between countries were not consistent. In light of this, it is important to further examine regional variation in diagnostic labeling and management practices among children with asthma as well as those without asthma but with conditions labels which may be used in place of asthma.

Our findings confirm those of global and regional investigations of childhood asthma prevalence where geographic variation has been observed [1–3, 11]. Generally, Westernized nations have experienced a higher prevalence of asthma than non-Westernized nations. While global differences have been relatively clear based on the ISAAC studies, more regionally, within Europe, differences have also been shown to exist. One previous study following ISAAC protocols found that there was higher wheeze prevalence in Scandinavian centres compared to Eastern European centers [11]. In addition to this, there was a gradient where the highest prevalence was observed in Scandinavia (Sweden and Finland; 11–19 %) followed by Estonia, Latvia, and Poland (7.5–8.5 %), countries with increasingly Westernized culture, and lowest in Albania, Romania, Russia, Georgia, and Uzbekistan (2.6–5.9 %), countries with the least amount of Western influence [11]. In a separate study of Eastern and Central Europe, similar patterns of asthma prevalence were observed with a high between-country variation but lower within-country variation in asthma prevalence [3]. However, from this study, it was suggested that diagnostic differences may explain some of the differences in asthma prevalence with bronchitis being used as a label more frequently in these centres compared to Western centres [3]. In our study, we advance this concept by showing that even within relatively close geographic proximity, these labeling differences occur where we see increased risk of asthma in Poland but a significant inverse association with spastic bronchitis in Poland.

Regardless of the outcome considered (asthma, wheeze in the past 12 months, or spastic bronchitis), consistent associations were observed personal and genetic factors. A parental history of allergic disease suggests a strong genetic component, which is well known to occur in asthma and allergic disease [12, 13]. Given that wheeze and spastic bronchitis are not thought to be as linked to allergic disease and family history as asthma, this provides some evidence that there may be some labelling issues, especially among those with spastic bronchitis given the similarity in odds ratio with asthma (2.72 vs. 3.01, respectively).

Associations between the environment and asthma have been less consistent. With regard to home mold or dampness, each association was statistically significant with a similar strength of association (odds ratios = 1.28–1.35). Mold and dampness has been associated with asthma and wheeze fairly consistently in recent years [14, 15]. While having a dog showed a consistent inverse association with each of the three outcomes considered, it was only statistically significant with spastic bronchitis and a trend towards statistical significance with asthma. However, the strength of each of these associations was almost identical (odds ratios = 0.78 vs 0.77). With the similarity in associations, it again adds some evidence that some children with spastic bronchitis may truly be children with asthma. Several other studies have reported a protective effect. However, these associations are not consistent and can be complex, often depending on other factors, and timing of exposure.

There was also an inverse association between age with wheeze in the past 12 months and age with ever diagnosed with spastic bronchitis. This association is easily explainable for wheeze in the past 12 months since a high proportion of children have wheeze while they are young but will grow out of this transient wheeze later in life. It is a little more difficult to explain the inverse association with spastic bronchitis. Given that the prevalence of this outcome is a cumulative indicator (ever diagnosed), we would expect that the risk of having this diagnosis would increase with age. Speculatively, it could be that the true prevalence of asthma is increasing but there is mislabelling where those with asthma are being labelled with spastic bronchitis. In younger children, there will be more labeling than had occurred with older children, leading to an inverse association.

The consistency between countries in the relationship between the various exposures and outcomes was encouraging and adds strength to our study. However, we did find that some of the associations were dependent on country. In general, while the associations were relatively consistent, results from the Ukraine were weaker and not statistically significant. A notable and consistent exception was that of home mold or dampness which showed significant effect modification with country regardless of outcome. In each case, associations in Belarus and Poland showed statistically significant increased risk while in Ukraine, non-significant inverse associations were observed. In a previous Canadian study, similar differences in effect were observed between two south Saskatchewan regions when considering mold and dampness [16]. While interesting, some caution must be taken since the results in Ukraine were weak and not statistically significant for this variable. However, differences in the housing or environment may be leading to different types of mould exposure, either in quantity or diversity, which may then be leading to differential associations with the outcomes. Those dwelling in Ukraine were much more likely to report home mold or dampness in the home compared to those in Belarus or Poland. A more rigorous investigation with objective measures would help explain these observations.

One of our research foci was to examine the association between rural living and asthma. We did not find statistically significant associations between rural residence and any of the considered outcomes. While not entirely consistent, previous research has suggested less asthma prevalence in rural areas compared to urban areas [17, 18] In our multivariate analyses we did not observe this association with asthma, wheeze in the past 12 months, or spastic bronchitis. In previous studies, farm dwelling has generally showed a stronger, more consistent, association with asthma than the more general rural dwelling. It may be that in our analyses, we did not capture the environmental effect associated with asthma through our definition of rural. Despite this, we found many differences in environmental exposures between urban and rural dwellers within each country and that based on crude analyses, the prevalence of diagnosed allergic disease and symptoms (e.g., eczema, rhinitis, general allergy) were almost always significantly higher in urban compared to rural dwellers, regardless of country. This is consistent with the existing literature which has shown more consistent associations with allergic disease than with asthma.

When we considered symptom characteristics among those with asthma by country, few consistent patterns emerged. One difficulty was that our statistical power in this phase of the analysis was greatly reduced. Despite this, among children with asthma, those living in Poland consistently had a lower likelihood of being diagnosed with spastic bronchitis or being hospitalized due to breathing problems but an increased risk of having ever been diagnosed with rhinitis. These differences may be related to better identification and labelling of asthma, avoiding terms such as spastic bronchitis, which may lead to better outpatient management. It is important to consider that in these comparisons, we are considering children who had a previous diagnosis of asthma and who were then possibly better managed, which makes interpretation of this part of the analysis difficult, explaining some of the inconsistency. In addition to this, the health care systems themselves may have a role in the diagnostic policies and management following a diagnosis, adding an extra layer of complexity to the interpretation of results comparing children with a previous diagnosis of asthma.

The current study has several strengths and limitations. First, this was a cross-sectional study, which limits our ability to investigate associations between risk factors and asthma. However, cross-sectional studies are ideally suited for the assessment of the prevalence of disease, which was a major focus of our study. The response rate was excellent in each region making reported prevalence representative of these populations, although not of the entire countries involved. A high response rate is necessary to complete studies of asthma prevalence. The sample size in each region was also high reducing the likelihood of low statistical power and Type II error. While we could not assess the temporality of the associations investigated, we used identical questionnaires and protocols in each of the regions while completing the study at the same point in time. In addition to this, our study allows us to consider the associations in a consistent manner between locations so that in the event of bias, it will be consistent across study locations. Asthma diagnosis lacks on objective gold standard and questionnaire report of a doctor’s diagnosis of asthma is used very frequently in epidemiological studies. This method has reasonable levels of agreement with other methods of assessment and has been suggested as the method of choice for large epidemiologic studies [19] given its validity and practical application. Third, this study was based on self-report and used a proxy reporter. This may result in some misclassification. However, previously, the sensitivity and specificity of this method to assess asthma has been high compared to blinded physician assessment [20]. Also, respiratory symptoms have been found to be accurately reported by the adolescent and strongly agree with parental reports in terms of asthma diagnoses [21]. Another possibility is that the participant experienced respiratory symptoms and did not seek medical attention. However, we examined differences in prevalence of symptoms in addition to diagnosed conditions, which should minimize biases due to labeling. Within Poland, an area included in the current study, we previously found high agreement between survey reported asthma and clinical evaluation of asthma among those children where asthma was already reported suggesting that asthma diagnosis is relatively accurate when applied but that among those without a label of asthma but with respiratory symptoms in the past year, there may be substantial underdiagnosis of asthma [22]. Additionally, language barriers may bias the study results. Efforts were made to ensure accurate translation by European members of the study team who each speak multiple languages, including those included in the study regions. Part of our analysis considered rural vs. urban comparisons. It is possible that the design we used did not truly capture the individual’s exposure based on rural settings. For example, if an urban dweller frequently visited a farm or had farm type exposures in town. There are many methods of defining rural [23] and we chose to use one that would capture exposures based on agricultural activity. Finally, potential confounding factors that were not addressed in the current analysis are the management practices and health care systems in each of the countries considered. The type and frequency of medication use may be dependent on differences in the healthcare system and asthma medication reimbursement procedures. According to our knowledge, in Ukraine, Belarus and Poland, children’s health care is free with open access to physicians. Also a high proportion of asthma medication is reimbursed. These problems are beyond the current publication and deserve a more detailed and separate analysis.

Future work in this area will include further investigation of diagnostic labeling and presenting patterns in the region. As part of this, work must be completed to assess and implement practice guidelines for the diagnosis and management of childhood asthma. This would include education and awareness programs aimed physicians and health care professionals as well as the general population. Results from Poland showing increases in asthma prevalence over a 21 year span [6] may serve as an example of changes that can occur after implementation of guidelines. While we were unable to directly link individual results with implementation of asthma consensus guidelines within countries, the timing allows us some interpretation linking the increases in asthma prevalence and improved asthma management Over this time, the proportion of those with asthma who were treated also improved [6] suggesting that these improvements could occur.

## Conclusion

In conclusion, we found that there was significant variation in asthma prevalence between the three BUPAS countries and that some of this variation is likely due to diagnostic differences between centres. By understanding patterns of geographic variation in asthma prevalence, we will be in a better position to study the etiology and better plan for asthma management.

## Abbreviations

ANOVA: Analysis of variance
BMI: Body mass index
BUPAS: Belarus, Ukraine, Poland Asthma Study
CI: Confidence Interval
cm: Centimeter
ISAAC: International study of asthma and allergies in childhood
Kg: Kilogram
OR: Odds ratio
p-value: Probability value
SOB: Shortness of breath
SPSS: Statistical package for the social sciences
